# A single mitochondrial DNA deletion accurately detects significant prostate cancer in men in the PSA ‘grey zone’

**DOI:** 10.1007/s00345-017-2152-z

**Published:** 2017-12-16

**Authors:** Jennifer Creed, Laurence Klotz, Andrew Harbottle, Andrea Maggrah, Brian Reguly, Anne George, Vincent Gnanapragasm

**Affiliations:** 1 MDNA Life Sciences UK Ltd, Newcastle upon Tyne, UK; 2 MDNA Life Sciences Inc, 2054 Vista Parkway, Suite 400, West Palm Beach, FL 33411 USA; 3 Division of Urology, Sunnybrook Health Sciences Centre, University of Toronto, Toronto, Canada; 4 Academic Urology Group, Department of Surgery and Oncology, University of Cambridge, Cambridge, UK; 5 Cambridge Urology Translational Research and Clinical Trials, Cambridge Biomedical Campus, Cambridge, UK

**Keywords:** Mitochondrial DNA, Prostate cancer, Multi-parametric magnetic resonance imaging, Plasma, Biomarker, DNA deletion, Real-time PCR, Diagnostic accuracy

## Abstract

**Purpose:**

To determine the clinical performance of a blood-based test for clinically significant (CS) prostate cancer (PCa) (grade group ≥ 2) intended for use in men with prostate serum antigen levels in the ‘grey zone’ (PSA < 10 ng/ml). The test quantifies a previously described 3.4 kb mitochondrial DNA (mtDNA) deletion.

**Methods:**

In a first prospective study of an MRI-guided re-biopsy population (*n* = 126), the 3.4 kb deletion and 18S rRNA gene were amplified from plasma. A diagnostic threshold was selected from the coordinates of the receiver operating characteristic curve and tested in a second population of men who were (*n* = 92) biopsy naïve when the mtDNA deletion was assayed and for whom those diagnosed with cancer on initial biopsy were treated with radical prostatectomy.

**Results:**

The 3.4 kb deletion was a good predictor of CS PCa in the image-guided re-biopsy population [AUC 0.84, (95% CI 0.73–0.95)] and the selected threshold corresponded to a sensitivity of 87% [95% CI, 70–96%], specificity of 68% [95% CI, 47–85%] and negative predictive value (NPV) of 97%. Applying this threshold to the second population showed this deletion to be a strong predictor of CS cancer [AUC 0.98, (95% CI 0.94–1.02)], independent of PSA or age [sensitivity 100% (95% CI, 93–100%), specificity 90% (95%CI 73–98%) and NPV 100%].

**Conclusion:**

The 3.4 kb deletion in plasma is an accurate predictor of CS cancer for men in the PSA ‘grey zone’. Used in advance of biopsy for improved patient selection, this deletion may reduce the number of biopsies needed to diagnose CS prostate cancers.

**Electronic supplementary material:**

The online version of this article (10.1007/s00345-017-2152-z) contains supplementary material, which is available to authorized users.

## Introduction

Indication for prostate biopsy due to suspicion of prostate cancer is primarily driven by elevated levels of serum prostate-specific antigen (PSA) [1, 2]. While inexpensive and accessible, effective use of PSA for screening is limited by its low diagnostic accuracy [3]. Further, most PSA measurements are within the PSA ‘grey zone’, < 10 ng/ml where accuracy is much lower [4]. Thus, the identification of a prostate cancer biomarker that can be measured in blood, provided at low cost, is independent of PSA, and accurately identifies CS prostate cancers that would benefit from near-term intervention, is a pressing need.

We and others have previously reported the clinical value of a 3.4 kb mitochondrial DNA (mtDNA) deletion (Online Resource 1) strongly correlated with the presence of prostate cancer and elevated even in normal-appearing prostate cells coexisting with malignant cells—exhibiting the concept of field cancerization [5–8]. Using laser capture micro-dissection, we reported that the quantity of the deletion while significantly elevated in malignant prostate cells, was not elevated in benign prostate conditions such as hyperplasia and inflammation [5]. As a biomarker derived from the mitochondrial genome, this deletion has several inherent biological traits that confer an advantage over its nuclear genome counterparts [9–11]. Briefly, mitochondrial genome deletions are heteroplasmic [9] and cellular energy production can be maintained even with a significant proportion of mutated mitochondrial genomes [9]. Thus, the mutated deleted species can persist in the cell instead of being repaired, increasing the window of time for detection. The subcellular location of mtDNA within the mitochondrial matrix positions it on the front line of cellular stress responses such as reactive oxygen species production, thus mtDNA mutation events can be an early indicator of the disease [10]. Mitochondrial alterations can contribute directly to tumour cell progression and metastasis, meaning, these mutations can be closely entwined with the disease itself [12]. Finally, mtDNA outnumber the nuclear genome in each cell by hundreds to thousands of copies, translating to robust detection in samples with even limited cellularity such as blood plasma [11].

Here we have focused on determining the diagnostic accuracy of the 3.4 kb mtDNA deletion in its cell-free circulating DNA form for men in the PSA ‘grey zone’ (PSA < 10 ng/ml) to detect CS cancer, defined as grade group 2 or greater (Gleason score 7 or greater). In this study, we defined a diagnostic threshold in an image-guided re-biopsy population and tested its performance in a second population of men who were biopsy naïve at the time of blood collection. Men diagnosed with prostate cancer in the second population were diagnosed on first biopsy and treated with radical prostatectomy, thus deletion levels were correlated to final surgical pathology.

## Methods

### Study cohorts

#### Image-guided re-biopsy population

Patients and samples were recruited from prostate cancer clinics at the Department of Urology, Cambridge University Hospitals Trust (CUHT), Cambridge UK [Ethics (Ethics 03/018—NRES Committee East of England, UK)]. All men previously had a PSA < 10 ng/ml, at least one prior negative prostate biopsy and required re-biopsy because of ongoing suspicion of cancer. Blood was collected for testing prior to MRI-guided repeat biopsy. All had a mpMRI and proceeded to trans-perineal template biopsy regardless of whether a target lesion was identified or not. Patients underwent mpMRI on a 1.5T MR450 or 3T Discovery MR750-HDx system (GE Healthcare, USA) with a multi-channel surface phased array coil, including standard anatomical and functional diffusion-weighted imaging using multiple b-values, as previously described [13]. T2W and DWI sequences were evaluated and scored using a Likert scale of cancer probability, based on the prostate imaging reporting and data system (PI-RADS) descriptors developed by the European Society of Urogenital Radiology (ESUR) [14]. The contours of mpMRI defined lesions (Likert 3–5) were drawn on the BiopseeTM fusion platform (Medcom, Germany). No lesion or Likert 1–3 lesions were considered mpMRI negative for this study. 24 sectoral biopsies + 2 from each target area using image fusion guidance (where applicable) were taken using a previously published standardized template [13]. Men without mpMRI lesions (mpMRI negative) had only 24 sectoral biopsies taken. Biopsy samples were processed through a single pathology laboratory and reported by specialist uropathologists (using ISUP 2005) [15].

#### First biopsy cohort

Patients and samples were recruited by the Ontario Tumour Bank (Toronto, Canada) and Bioreclamation IVT (Westbury, New York, USA). All men were biopsy naïve at the time of blood collection. For cancer positive cases the inclusion criteria were (1) men diagnosed with cancer on first biopsy (2) presenting with PSA < 10 ng/ml, (3) plasma collected pre-biopsy and (4) treated with a radical prostatectomy with final surgical histology available. Age at the time of blood collection was recorded as within 5-year intervals, thus for analysis, the median value of the interval was used. Biological materials were provided by the Ontario Tumour Bank. As control (non-cancer) samples were not available from the Ontario Tumour Bank, male control samples were obtained from Bioreclamation IVT. PSA levels were not available for these men, however, at the time of enrolment none had a prior diagnosis of prostate cancer and were not in a urologist’s care due to suspicion of prostate cancer as verified during donor screening by Bioreclamation IVT.

### Sample handling and processing

Blood samples were collected in K2EDTA and plasma was separated by centrifugation and frozen until DNA extraction. DNA was isolated from 200 μl plasma using the QIAamp 96 QIAcubeHT extraction kit (Qiagen, Crawley, UK). The 18S rRNA gene and 3.4 kb deletion were quantified using quantitative polymerase chain reaction. Detailed methods and assay performance are provided in Online Resource 2.

*PCR reagent kit*–The PCR mixture, positive controls, and primers for both 18S rRNA and the 3.4 kb deletion for detection in DNA extracted from blood plasma will be available as a Research Use Only (RUO) reagent kit in 2018. This will allow any third party to continue independent investigations using the same high-quality reagents and optimized assay parameters established by MDNA. The availability of the RUO reagent kit is intended to achieve better quality control and limit interlaboratory assay variability.

### Statistical analysis

All samples and targets were amplified in triplicate, and average Cq values were calculated. The normalised 3.4 kb deletion value (ΔCq) was determined by relative quantification of the 3.4 kb deletion to the 18S rRNA reference gene. Statistical analyses were performed using Graphpad Prism 5.0, (Graphpad software Inc, La Jolla, CA, USA) for receiver operating characteristic (ROC) curves and descriptives. SPSS v.17.0 (IBM Corp., Armonk, NY, USA) was used for all correlations and tests of significance. The clinical characteristics were summarized using count and percentages for the categorical data, and the mean, standard deviation, and range for continuous variables. The means of two groups were compared using the Student’s *t* test and the Mann–Whitney *U* test for parametric and non-parametric distributions, respectively. The correlation between the two variables was assessed with the Pearson correlation coefficient (*r*). With respect to the presence of CS prostate cancer, ROC curves were constructed for the 3.4 kb mtDNA deletion. The area under the curve (AUC) of the ROC and the sensitivity and specificity at selected cut-offs were calculated with 95% confidence intervals (95% CI). A *p* value < 0.05 was considered statistically significant for all tests. For men in the CUHT cohort, the final pathological outcome was based on the image-guided re-biopsy, where men with two negative biopsies and a negative MRI were considered negative controls (MRI-/Bx-). For men in the biopsy naive cohort, outcome was based upon grade group determined at prostatectomy. Men in the control group did not have surgical outcomes and were considered negative due to the absence of clinical suspicion of prostate cancer.

## Results

### Performance of the 3.4 kb mtDNA deletion in an MRI-guided re-biopsy population

#### Patient characteristics

The clinical and demographic characteristics of the 126 participants in this cohort are summarized in Online Resource 3. Seventy-six (60.3%) had a diagnosis of prostate cancer at the close of the study, with 31 (24.6%) having CS cancer. Of men without a diagnosis of cancer, 25 (50%) were both histologically and MRI negative [MRI (−)/Bx(−)]. The mean (± s.d.) age for all patients, all cancer patients, all CS cancer patients, and all MRI(-)/Bx(−) controls was 63.77 (± 6.55), 64.51 (± 6.18), 65.10 (± 5.90), 61.80 (± 7.57), respectively. The mean (± s.d.) PSA for all patients, all cancer patients, all CS cancer patients, and all MRI(−)/Bx(−) controls was 6.38 (± 4.09), 6.63 (± 4.93), 5.78 (± 3.37), 6.21 (± 2.30), respectively. The difference between groups for age and PSA was not statistically significant.

#### 3.4 kb mitochondrial DNA deletion quantity for prediction of CS cancers

**Fig. 1:**
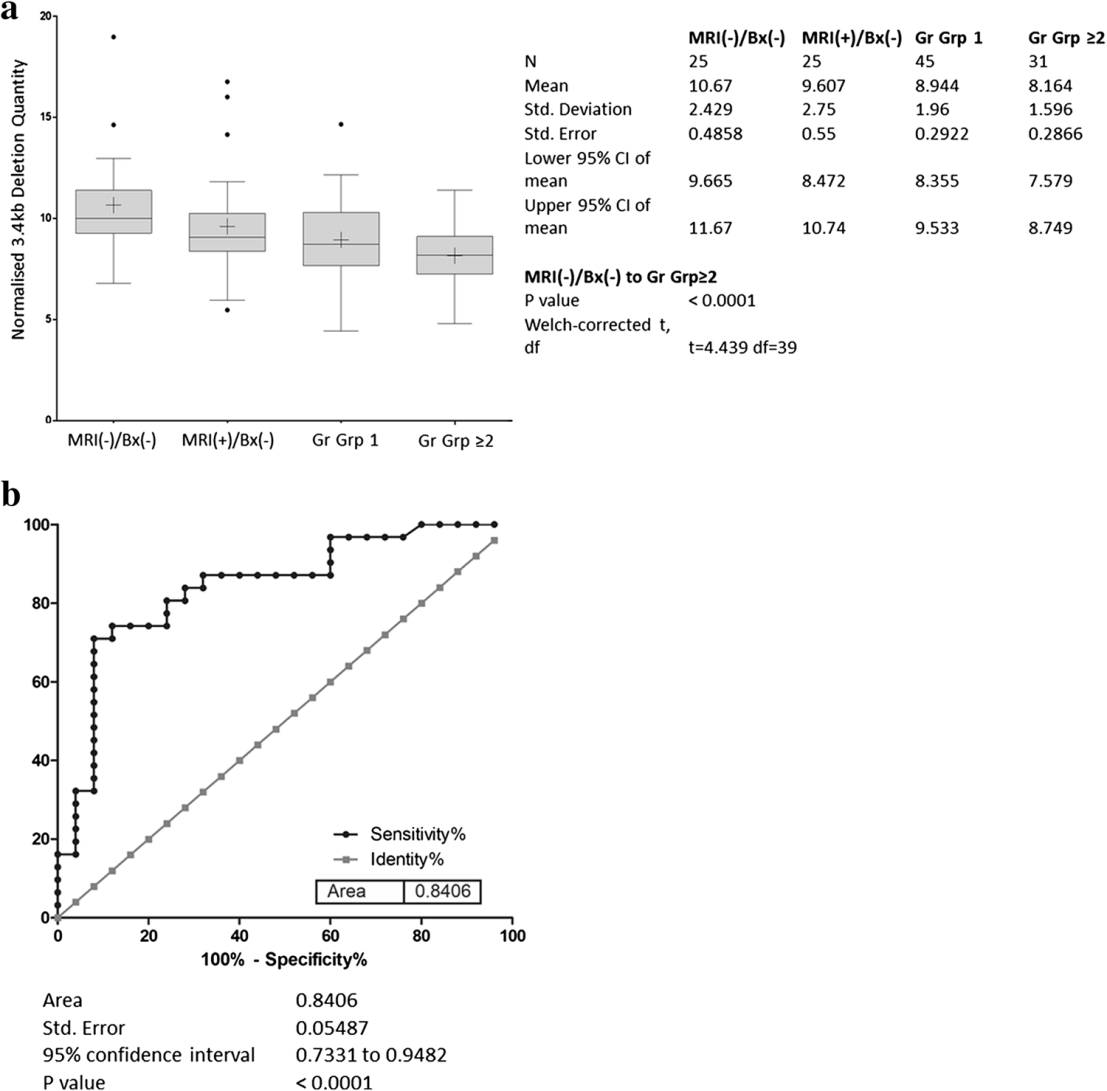
**a** Distribution of normalised 3.4 kb mitochondrial DNA deletion values for each clinical subgroup in the repeat biopsy cohort. The horizontal line within the box indicates the median, boundaries of the box indicate the 25th and 75th percentile, and the whiskers indicate 10th and 90th percentile of the results. The “+” marked in the box indicates the mean. *Gr Grp* grade group. **b** Receiver operating characteristic curve of the 3.4 kb deletion in the repeat biopsy cohort. Normalised 3.4 kb mtDNA deletion in blood plasma from men in the PSA ‘grey zone’ showing the accuracy of detection of CS (Gr Grp ≥ 2) cancers

The distribution of the 3.4 kb deletion ($\Delta {\text{Cq}}$) among MRI-/Bx- control samples, MRI +/Bx-, GS6 and CS cancers is shown in Fig. 1a. The mean (± s.d.) $\Delta {\text{Cq}}$ for control samples was 10.67 (± 2.429), while the mean (± s.d.) $\Delta {\text{Cq}}$ for CS PCa patients was 8.16 (± 1.96). This difference was significant at *p* < 0.0001. 3.4 kb deletion levels in the MRI +/ Bx- and grade group 1 prostate cancers were both intermediate between the controls and CS cancers with means of 9.61 (± 2.75) and 8.94 (± 1.96) respectively. The nuclear DNA 18S rRNA gene used for normalisation of the deletion quantity did not vary significantly between groups (*p* = 0.170) with a mean (± s.d.) 25.5 (± 1.627) for controls and mean (± s.d.) 26.01 (± 0.914) for CS cancers. Neither PSA nor age were significantly correlated with 3.4 kb deletion quantity, *r* = − 0.052 and 0.038, respectively. The 3.4 kb deletion for detection of CS cancers (*n* = 31) produced an AUC of 0.84 [95% CI, 0.73–0.95], *p* < 0.0001, when compared to levels detected in the control population (*n* = 25) (Fig. 1b). Using the ROC coordinates a threshold of 9.68 was selected to maximize the number of CS cancers detected while maintaining acceptable specificity and corresponded to a sensitivity of 87% [95% CI, 70–96%], and a specificity of 68% [95% CI, 47–85%].

#### 3.4 kb mitochondrial DNA deletion for prediction of any cancer

The selected diagnostic threshold was applied to determine the accuracy with which participants diagnosed with any grade group prostate cancer could be distinguished from the control group. This yielded an AUC 0.77 [95% CI, 0.67–0.87], *p* < 0.0001 with comparable specificity to the CS cancer at 68% [95% CI, 46–85%] but lower sensitivity at 75% [95% CI, 64–84%], meaning more grade group 1 cancers than CS cancers are considered negative at the selected cut-off.

#### 3.4 kb mtDNA deletion and MRI

As all men in this cohort were subject to mpMRI, the accuracy with which the 3.4 kb deletion could predict an outcome of CS cancer on subsequent biopsy was compared with that of mpMRI using both dichotomous classification of lesion positive or lesion negative, as well as Likert Score. When an indication for biopsy was determined as the detection of a suspicious lesion mpMRI identified 86% of CS cancers with a specificity of 50%; when restricting indication for biopsy to a Likert Score of 4 or 5 the sensitivity decreased to 74% with an increase in specificity to 76%. In the subgroup of men with a negative MRI (*n* = 71), defined as no suspicious lesion, the 3.4 kb deletion appeared predictive of CS cancers [AUC 0.82, (95% CI, 0.65–0.98), *p* < 0.0001]. At the defined threshold of 9.68, the sensitivity was 88% [95% CI, 47–100%] and specificity was 53% [95% CI, 36–69%].

### Validation of the 3.4 kb mitochondrial DNA deletion performance in a first biopsy cohort

#### Patient characteristics

The clinical and demographic characteristics for the 92 participants of this cohort are summarized in Online Resource 3. Sixty-three (68.5%) had an outcome of prostate cancer and 47 (51.1%) were CS prostate cancer. The mean (± s.d.) age for no cancer controls, those with any cancer, and those with CS cancers were 51.83 (± 4.49), 62.63 (± 5.60), and 62.53 (± 5.86), respectively. Men in the control group were significantly younger than the men with a diagnosis of cancer (*p* < 0.0001). The mean (± s.d.) PSA for men with any cancer and those with CS cancers was 5.84 (± 2.22) and 6.01 (± 2.14), respectively, this difference was not statistically significant (*p* = 0.69).

#### 3.4 kb mitochondrial DNA deletion quantity for prediction of CS cancers

**Fig. 2:**
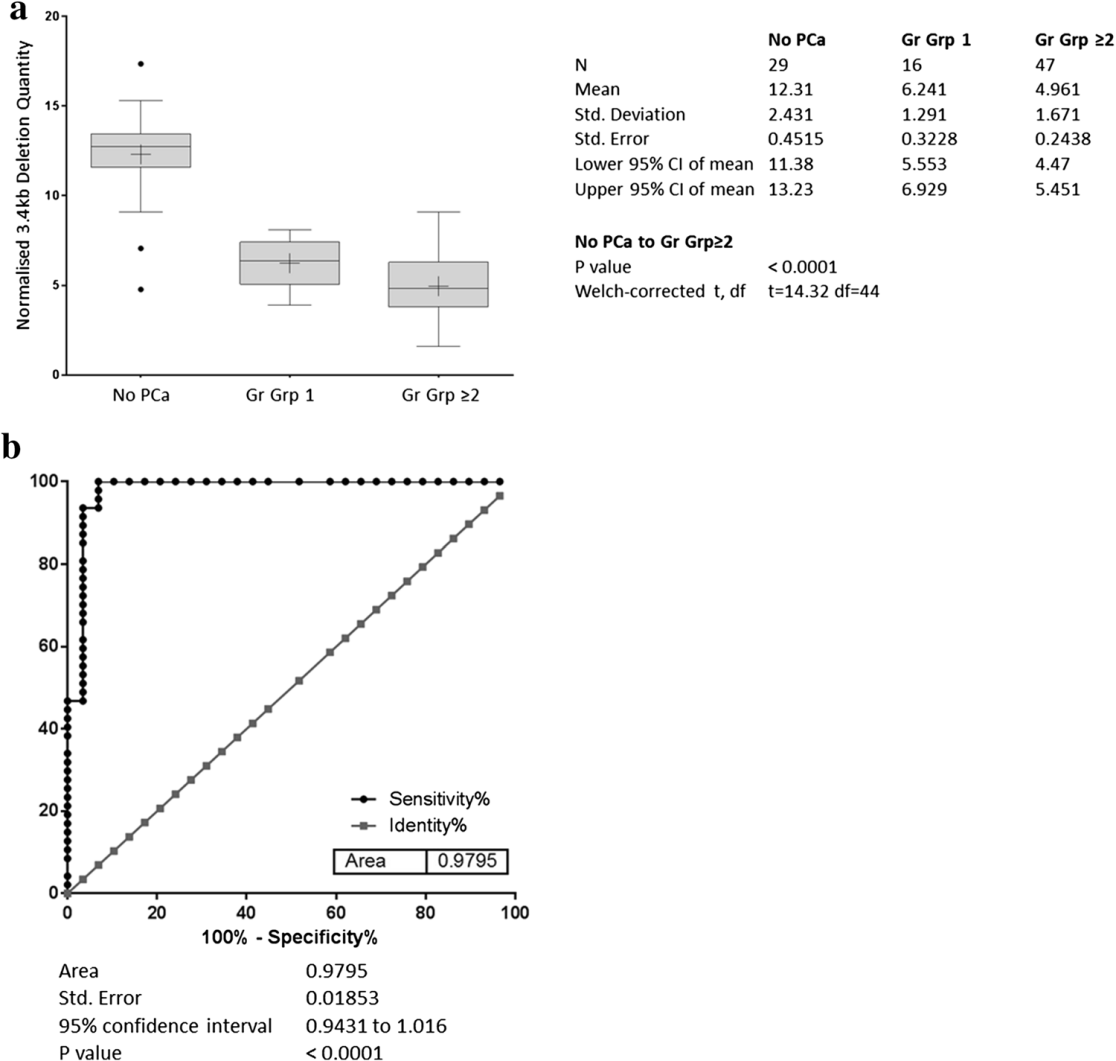
**a** Distribution of the 3.4 kb deletion in the first biopsy cohort. The horizontal line within the box indicates the median, boundaries of the box indicate the 25th and 75th percentile, and the whiskers indicate 10th and 90th percentile of the results. The “+” marked in the box indicates the mean. *Gr Grp* grade group. **b** Receiver operating characteristic curve of the 3.4 kb deletion in the first biopsy cohort. Normalised 3.4 kb mtDNA deletion in blood plasma from men in the PSA ‘grey zone’ showing the accuracy of detection of CS (Gr Grp ≥ 2) prostate cancers

The distribution of the 3.4 kb deletion for each clinical group is shown in Fig. 2a. The mean (± s.d.) $\Delta {\text{Ct}}$ for control samples was 12.31 (± 2.43), for grade group 1, 6.24 (± 1.29), and for CS prostate cancer patients, 4.96 (± 1.67). This difference in deletion quantity between controls and CS cancer was significant at *p* < 0.0001. Figure 2b shows the diagnostic accuracy of the 3.4 kb deletion, AUC 0.98 [95% CI, 0.94–1.02], *p* < 0.0001. Applying the threshold of 9.68 for discrimination of CS cancers corresponds to a sensitivity of 100% [95% CI, 93–100%] and a specificity of 90% [95% CI, 73–98%]. This threshold correctly classified all 47 CS cancers and 25 of the 29 controls. All 16 men with grade group 1 cancers in this cohort were classified at this threshold as positive. The NPV and positive predictive value (PPV) were calculated at both the CS cancer incidence rate of the cohort of 62% as well as downward adjusted to 10%, a figure more consistent with the incidence observed in practice in the intended use population [16]. At both incidence levels, the NPV was 100% as all CS cancers were correctly classified with the deletion. The PPV was 92.1% and 44.25% for the two incidence levels, respectively. No significant correlation was observed between the 3.4 kb deletion and either PSA (*r* = − 0.098) or age (*r* = 0.104).

## Discussion

This study is the first to report the detection of the 3.4 kb mitochondrial DNA deletion in plasma and establish and validate a useful diagnostic threshold for the accurate detection of CS prostate cancer for men in the PSA ‘grey zone’. Prior reports describe the detection of the 3.4 kb deletion in prostate tissue, serum and urine and its correlation with prostate cancer [5–7]. However, assay conditions defined for tissue were not optimal for detection in fluids, resulting in an unacceptable failure rate [6]. To optimize detection for use in blood plasma, we reduced the amplicon size for both the 3.4 kb deletion and the internal control, normalised input DNA using the internal control rather than DNA concentration determined by absorbance at 260 nm, and avoided the use of wild-type mitochondrial DNA as it is widely reported to vary significantly with many other cancers as well as aging [17, 18] and was expected to negatively impact specificity for prostate cancer. With these optimizations, all samples in this study generated results meeting the defined quality requirements of the assay.

The diagnostic accuracy of the 3.4 kb deletion in plasma was determined first in an image-guided repeat biopsy cohort [AUC 0.84 (95% CI, 0.73–0.95), *p* < 0.0001]. A threshold of 9.68 ∆Cq was selected from the coordinates of the curve that corresponded to a sensitivity of 87% [95% CI, 70–96%], specificity of 68% [95% CI, 47–85%] and NPV of 97% for detection of CS cancers. We chose to select the threshold in the repeat biopsy population as the cohort was exceptionally well qualified with both histological and radiological confirmation of disease status. As the gold standard of biopsy is associated with a significant false negative rate [19, 20], selection of an optimal diagnostic threshold in a control group defined by histology alone can be expected to include 30–60% or more undetected cancers [19, 20]. Thus, determination of the diagnostic threshold in the well-qualified image-guided repeat biopsy cohort reduced this risk [21].

The defined threshold was then tested against a second population of men who were biopsy naïve at the time of plasma collection. In this cohort, men with prostate cancer were diagnosed on first rather than repeat biopsy and were treated surgically with radical prostatectomy. Performance of the 3.4 kb deletion using the defined threshold was very high, sensitivity of 100% [95% CI, 93–100%], specificity of 90% [95% CI, 73–98%] and NPV of 100%. Thus, the same diagnostic threshold was effective for the identification of CS cancers in either the repeat biopsy or first biopsy setting though more robust in the latter.

Interestingly, the mean quantity of the 3.4 kb deletion was significantly higher in men diagnosed on first biopsy compared to the repeat biopsy cohort when stratified by grade group, such that grade group 1 cancers detected on first biopsy had a significantly greater quantity of deletion than did grade group 1 cancers detected on repeat biopsy. The same finding was true for CS cancers. This may suggest that the increase of the 3.4 kb deletion may be associated not only with decreasing differentiation as seen between well differentiated (grade group 1) and poorly differentiated cancers (grade groups 2+) but perhaps tumour volume as tumours diagnosed on first biopsy are reportedly larger than those diagnosed on repeat biopsy [22, 23].

Compared to other biomarkers of prostate cancer, the 3.4 kb deletion in a liquid biopsy format has significant advantages. Blood plasma is a common, non-invasive sample matrix amenable to routine testing, and a DNA analyte is more stable than other classes of molecules such as micro-RNAs [24]. The performance of the biomarker, independent of PSA, supports its use for any man with total PSA in the ‘grey zone’ who is being considered for biopsy, in contrast to PSA-derived biomarkers which may be contraindicated or require adjustment for men using 5-alpha-reductase inhibitors [25, 26]. The standard DNA extraction, qPCR process and result of this single-biomarker assay is simple and cost-effective. The assay itself is robust with a negligible failure rate. Finally, the accuracy with which CS prostate cancer within the PSA ‘grey zone’ is correctly identified or ruled out appears to be unsurpassed by other liquid biopsy biomarkers such as %fPSA [AUC 0.59 (95% CI, 0.53–0.65)], *phi* [AUC 0.64 (95% CI, 0.59–0.71)], %p2PSA [AUC 0.64 (95% CI, 0.58–0.70)] [27], and PCA3 [AUC 0.71 (95% CI, 0.61–0.81)] [28]. (It should be noted that the lower limit of PSA ‘grey zone’ varied between these analyses, specifically 2 ng/ml for %fPSA, *phi*, and %p2PSA, and 4 ng/ml for PCA3).

In practice, though PSA is a good indicator of prostate cancer at significantly elevated levels (> 10 ng/ml) only a small proportion of all PSA tests performed for screening purposes are above this cut-off [16]. Additionally, there is no lower cut-off for PSA beneath which CS cancers do not occur—up to 15% of men with PSA less than 4 ng/ml have CS prostate cancer [29]. This results in a significant number of men in the PSA ‘grey zone’, with a PSA < 10 ng/ml, and a poorly qualified risk of CS prostate cancer. The diagnostic uncertainty of this group results in a high proportion of unnecessary biopsies and thus a high NPV is of paramount importance for any biomarker intended to better qualify risk in this setting. Ideally, a negative biomarker result must correlate with the absence of CS cancer nearly perfectly. The NPVs reported here (97% in the repeat biopsy setting, and 100% in the first biopsy setting) appear to fulfil this requirement well, offering the potential to safely avoid a great many unnecessary biopsies while missing very few CS cancers.

As the 3.4 kb deletion performed with somewhat better accuracy than mpMRI in this study and was informative for men with a negative mpMRI result, this suggests the deletion may have additional promise complementing the use of mpMRI, however, this requires further study.

The main limitations of this study are the availability of well-qualified control samples for the first biopsy cohort, the use of grade group classifications as a surrogate for clinically significant cancer and the modest sample size.

## Conclusion

The 3.4 kb mitochondrial DNA deletion measured in blood plasma accurately predicts CS prostate cancer and correlates well to both image-guided biopsy outcome in a repeat biopsy setting as well as prostatectomy outcome in a first biopsy setting for men in the PSA ‘grey zone’. As a single biomarker independent of PSA, the test is simple, and the result represents novel information with respect to risk.

## Electronic supplementary material

Below is the link to the electronic supplementary material.

Supplementary material 1 (PDF 225 kb)

Supplementary material 2 (PDF 78 kb)

Supplementary material 3 (PDF 180 kb)
