# Supplementary material for: A single mitochondrial DNA deletion accurately detects significant prostate cancer in men in the PSA ‘grey zone’
Source: World J Urol. 2017 Dec 16;36(3):341–8. doi: 10.1007/s00345-017-2152-z (PMC5846823; doi:10.1007/s00345-017-2152-z)
Supplement: Supplementary file 1 — Supplementary material 1 (PDF 225 kb) [file 345_2017_2152_MOESM1_ESM.pdf]

Schematic representation of the human mitochondrial genome with the 3.4kb deletion breakpoints.

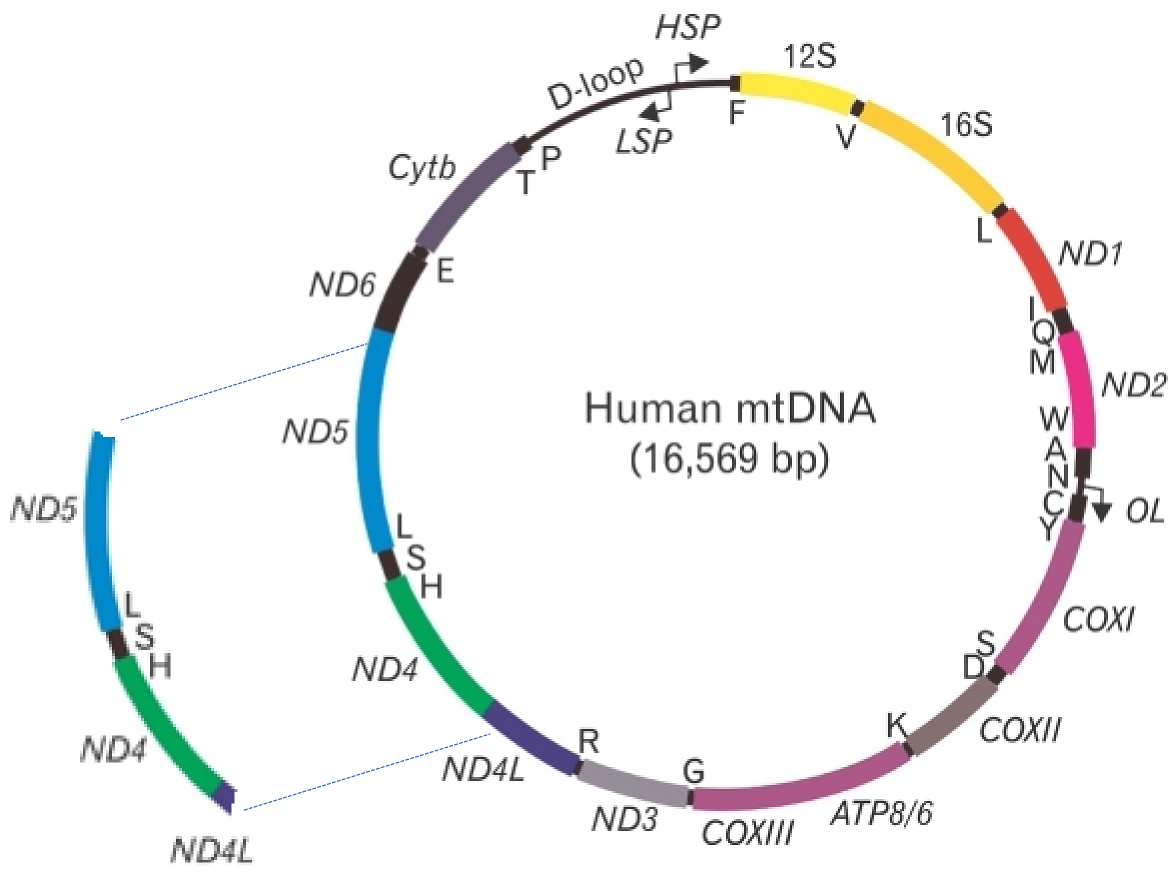

3.4 kb deletion
Breakpoint 10,744:14,124

Copyright Policy - Open Access License

A single mitochondrial DNA deletion accurately detects significant prostate cancer in men in the PSA 'grey zone'. World Journal of Urology. Jennifer Creed*, Laurence Klotz, Andrew Harbottle, Andrea Maggrah, Brian Reguly, Anne George, and Vincent Gnanapragasm

*Corresponding author:
MDNA Life Sciences
Email: j.creed@mdnalifesciences.com
