# Supplementary material for: A single mitochondrial DNA deletion accurately detects significant prostate cancer in men in the PSA ‘grey zone’
Source: World J Urol. 2017 Dec 16;36(3):341–8. doi: 10.1007/s00345-017-2152-z (PMC5846823; doi:10.1007/s00345-017-2152-z)
Supplement: Supplementary file 2 — Supplementary material 2 (PDF 78 kb) [file 345_2017_2152_MOESM2_ESM.pdf]

Detailed Methods

DNA Extraction

DNA was extracted from 200 µl of plasma using the QIAamp 96 QIAcubeHT extraction kit (Q*iagen, Crawley, UK*), automated on a QIAcube HT system (*Qiagen, Crawley, UK*). From 200 µl of plasma, 200 µl of purified DNA was eluted from the vacuum plate in AE buffer.

3.4kb mtDNA Deletion Real-time qPCR

Amplification reactions were performed as 20 µl reactions in a 96-well hard-shell white microplate *(Bio-Rad, Hemel Hempstead, UK).* Each well contained 5 µl of un-normalised DNA, 1X SYBR Green Mastermix, 250 nM of each primer. Primer sequences and mastermix are trade secrets of MDNA Life Sciences Inc but will be made available for Research Use Only in 2018. PCR and SYBR Green I fluorescence analysis was performed using a Chromo4 qPCR system *(Bio-Rad, Hemel Hempstead, UK)*. Amplification conditions were: 3 minutes at 95°C, followed by 45 cycles of 30 seconds at 95°C, 30 seconds at 65.5°C, and 30 seconds at 72°C. Following amplification melting curve analysis was performed from 70°C-90°C, reading every 0.5°C. Each plate of samples and controls was amplified in triplicate on three separate occasions.

Real-time qPCR Normalisation with 18s rRNA

Normalisation of the 3.4kb deletion quantity was carried out using the internal nuclear DNA control gene 18s rRNA.

Amplification reactions were performed as 20 µl reactions in a 96-well hard-shell white microplate *(Bio-Rad, Hemel Hempstead, UK).* Each well contained 5µl of un-normalised DNA, 1X SYBR Green Mastermix, 150 nM of each primer. Primer sequences and mastermix are trade secrets of MDNA Life Sciences Inc. PCR and SYBR Green I fluorescence analysis was performed using a Chromo4 qPCR system *(BioRad, Hemel Hempstead, UK)*. Amplification conditions were: 3 minutes at 95°C, followed by 40 cycles of 30 seconds at 95°C, 30 seconds at 64°C, and 30 seconds at 72°C. Following amplification melting curve analysis was performed from 70°C-90°C, reading every 0.5°C.

Quality Control

The quantification cycle (Cq) was calculated using the built-in regression model within the CFX manager software *(Bio-Rad, Hemel Hempstead, UK).* Cq of the 3.4kb mtDNA deletion was normalised to the Cq of the multi-copy nuclear target 18s. All samples were amplified in triplicate on separate plates, and considered to have passed if at least two of the replicates were within 1.5 Cq and the melting temperature (Tm) was consistent with the target amplification product when present, (3.4kb deletion Tm 81°C ± 2°C, 18s rRNA Tm 82°C ± 2°C).

Controls

Two negative template controls were processed alongside each batch of plasma DNA extractions and verified negative for amplification of both the 3.4kb deletion and the 18s rRNA sequence. Two negative template controls were processed with each PCR plate and verified negative for amplification of both the 3.4kb deletion and the 18s rRNA sequence.

Standards

Calibration curves were created using synthetic G-block sequences of the 3.4kb deletion and the 18s rRNA gene (Integrated DNA Technologies, Iowa, USA). Beginning with a 1:100 dilution of the stock G-block preparation, 10-fold serial dilutions over 6 orders of magnitude were created and amplified for either the 3.4 kb deletion or 18s rRNA. The correlation coefficient for both the 3.4kb deletion and the 18s rRNA sequences was 0.999.

A single mitochondrial DNA deletion accurately detects significant prostate cancer in men in the PSA 'grey zone'. World Journal of Urology. Jennifer Creed*, Laurence Klotz, Andrew Harbottle, Andrea Maggrah, Brian Reguly, Anne George, and Vincent Gnanapragasm

*Corresponding author:
MDNA Life Sciences
Email: j.creed@mdnalifesciences.com
