# Supplementary material for: A single mitochondrial DNA deletion accurately detects significant prostate cancer in men in the PSA ‘grey zone’
Source: World J Urol. 2017 Dec 16;36(3):341–8. doi: 10.1007/s00345-017-2152-z (PMC5846823; doi:10.1007/s00345-017-2152-z)
Supplement: Supplementary file 3 — Supplementary material 3 (PDF 180 kb) [file 345_2017_2152_MOESM3_ESM.pdf]

Online Resource 3, Table 1a. Image-guided repeat biopsy cohort clinical characteristics,

Clinical Characteristics	
N	126
Age (mean, range, SD)	63.77, 44-78, 6.55
Index PSA (mean, SD)	6.38, 4.09
Grade Group 1 (Count, %)	45, 35.7%
Grade Group 2 (Count, %)	21, 16.7%
Grade Group 3 (Count, %)	5, 4.0%
Grade Group 4 (Count, %)	2, 1.6%
Grade Group 5 (Count, %)	3, 2.4%
All cancers (count, %)	76, 60.3%
Grade Group ≥2 (count, %)	31, 24.6%
MRI(-),Bx(-) (count, %)	25, 19.8%
MRI(+),Bx(-) (count, %)	25, 19.8%

SD: standard deviation; PSA: prostate specific antigen; MRI: magnetic resonance imaging

Online Resource 3, Table 1b. Image-guided repeat biopsy cohort Comparison of clinical characteristics; all cancers, CS cancers and controls.

	All controls	All cancers	p value	CS cancers	p value
Patients, n	25	76	-	31	-
Age, yr, mean, SD	61.80, 7.57	64.51, 6.18	0.0755*	65.10, 5.90	0.0722**
PSA, ng/ml, mean, SD	6.21, 2.30	6.63, 4.93	0.6823*	5.78, 3.37	0.5890**

*p value for comparison to control group; **p value for comparison between all cancers and CS cancers

Online Resource 3, Table 2a. First biopsy cohort, clinical characteristics

Clinical Characteristics	
N	92
Age (mean, range, SD) for PCa Cases	62.63, 47-72, 5.60
Age (mean, range, SD) for Controls	51.83, 45-60, 4.49
PSA (mean, range, SD) PCa Cases	5.84, 1.20-9.90, 2.22
Grade Group 1 (Count, %)	16, 17.4%
Grade Group 2 (Count, %)	20, 21.7%
Grade Group 3 (Count, %)	19, 20.7%
Grade Group 4 (Count, %)	5, 5.4%
Grade Group 5 (Count, %)	3, 3.3%
All cancers (count, %)	63, 68.5%
Grade Group ≥2 (count, %)	47, 51.1%
Controls (count, %)	29, 31.5%

SD: standard deviation; PSA: prostate specific antigen; MRI: magnetic resonance imaging

Online Resource 3, Table 2b. First biopsy cohort, comparison of clinical characteristics; all cancers, CS cancers and controls

	All controls	All cancers	p value	CS Cancers	p value
Patients, n	29	63		47	
Age, yr, mean, SD	51.83, 4.50	62.63, 5.60	<0.0001*	62.53, 5.86	<0.0001**
PSA, ng/ml, mean, SD	NA	5.84, 2.22		6.01, 2.14	0.69**

*p value for comparison to control group; **p value for comparison between all cancers and CS cancers

A single mitochondrial DNA deletion accurately detects significant prostate cancer in men in the PSA ‘grey zone’. World Journal of Urology. Jennifer Creed*, Laurence Klotz, Andrew Harbottle, Andrea Maggrah, Brian Reguly, Anne George, and Vincent Gnanapragasm
*Corresponding author:
Jennifer Creed
MDNA Life Sciences
Email: j.creed@mdnalifesciences.com
